# Protocol for a scoping review of health equity frameworks and models applied in empirical studies of chronic disease prevention and control

**DOI:** 10.1186/s13643-023-02240-2

**Published:** 2023-05-11

**Authors:** Callie Walsh-Bailey, Amanda Gilbert, Thembekile Shato, Brittney Sandler, Ana A. Baumann, Cory D. Bradley, Gabriella M. McLoughlin, F. Hunter McGuire, Meredith P. Fort, Rachel G. Tabak

**Affiliations:** 1 Brown School, Washington University in St. Louis, 1 Brookings Dr, St. Louis, MO 63130 USA; 2 Implementation Science Center for Cancer Control and Prevention Research Center, Washington University in St. Louis, One Brookings Drive, St. Louis, MO 63130 USA; 3 Division of Public Health Sciences, Department of Surgery, Washington University School of Medicine, 600 S. Taylor Ave, St. Louis, MO 63110 USA; 4 Division of Infectious Diseases, Washington University School of Medicine, 600 S. Taylor Ave, St. Louis, MO 63110 USA; 5 Bernard Becker Medical Library, School of Medicine, Washington University in St. Louis, St. Louis, USA; 6 College of Public Health, Temple University, 1800 N. Broad St, Philadelphia, PA 19121 USA; 7 Department of Health Systems, Management and Policy, Colorado School of Public Health, 13055 E. 17Th Ave, Aurora, CO 80045 USA

**Keywords:** Models, Frameworks, Health equity, Scoping review, Chronic disease, Noncommunicable disease, Implementation science

## Abstract

**Background:**

Chronic diseases, such as cancers and cardiovascular diseases, present the greatest burden of morbidity and mortality worldwide. This burden disproportionately affects historically marginalized populations. Health equity is rapidly gaining increased attention in public health, health services, and implementation research, though many health inequities persist. Health equity frameworks and models (FM) have been called upon to guide equity-focused chronic disease and implementation research. However, there is no clear synthesis of the health equity FM used in chronic disease research or how these are applied in empirical studies. This scoping review seeks to fill this gap by identifying and characterizing health equity FM applied in empirical studies along the chronic disease prevention and control continuum, describing how these FM are used, and exploring potential applications to the field of implementation science.

**Methods:**

We follow established guidance for conducting scoping reviews, which includes six stages: (1) identify the research question; (2) identify relevant studies; (3) select studies for inclusion; (4) data extraction; (5) collating, summarizing, and reporting the results; and (6) consultation. This protocol presents the iterative, collaborative approach taken to conceptualize this study and develop the search strategy. We describe the criteria for inclusion in this review, methods for conducting two phases of screening (title and abstract, full text), data extraction procedures, and quality assurance approaches taken throughout the project.

**Discussion:**

The findings from this review will inform health-equity focused chronic disease prevention and control research. FM identified through this review will be added to an existing website summarizing dissemination and implementation science frameworks, and we will offer case examples and recommendations for utilizing a health equity FM in empirical studies. Our search strategy and review methodology may serve as an example for scholars seeking to conduct reviews of health equity FM in other health disciplines.

**Systematic review registration:**

Open Science Framework Registration 10.17605/OSF.IO/SFVE6

**Supplementary Information:**

The online version contains supplementary material available at 10.1186/s13643-023-02240-2.

## Background

Numerous intergovernmental organizations, national governments, professional societies, and other institutions recognize health and wellbeing as a fundamental human right [1]. Despite widespread recognition of the right to health, inequity persists as a critical public health issue globally. Power, wealth, and resources are not distributed equally across society; moreover, this distribution is not random, rather is the result of historical and modern injustices [2]. These unjust social, economic, and political structures lead to forms of systemic discrimination, marginalization, and oppression, such as racism, colonialism, sexism, heterosexism, ableism, xenophobia, and deny access to opportunities to achieve optimal health [2–4]. The goal of health equity work is to identify and ameliorate these injustices, and to ensure the necessary conditions for all people to live their healthiest lives [5]. There are multiple definitions of health equity; for conceptual consistency, we utilize the following definition: “Health equity means that everyone has a fair and just opportunity to be as healthy as possible. This requires removing obstacles to health such as poverty, discrimination, and their consequences, including [disenfranchisement] and lack of access to good jobs with fair pay, quality education and housing, safe environments, and health care” [6]. Expanding this definition, we follow Jones and colleagues’ conceptualization of health equity as a *process* that requires continuous action to address historical and contemporary injustices and to allocate resources according to need [5].

The need for equity-focused research and practice is ubiquitous across all areas of health, including chronic diseases (also referred to as non-communicable diseases). Chronic diseases present the greatest burden of morbidity and mortality worldwide, and disproportionately affect historically and intentionally excluded populations, including marginalized racial, ethnic, and gender identities, people ascribed low-socioeconomic position, and people who hold multiple of these or other intersecting marginalized identities [7, 8]. Seven of the 10 leading causes of death globally are chronic diseases; in 2019, these types of conditions, which include cardiovascular disease, cancers, diabetes, and chronic respiratory conditions, were responsible for nearly 75% of all deaths worldwide [9, 10]. Although there are numerous effective interventions for preventing, managing, and treating chronic diseases, these have been primarily developed and tested in well-resourced settings among predominantly white populations [11, 12]. Despite advancements in multi-level interventions, including policies aimed to modify structural influences on health, equity is not always a specific focus of their evaluation, consequently leaving the impact of structural interventions understudied [13, 14]. Moreover, for interventions that show promise to promote health equity and improve health outcomes, there is comparatively little evidence demonstrating *how* best to adopt, implement, and sustain them [15, 16].

The relatively new field of implementation science, which aims to reduce quality gaps and improve health outcomes through enhancing the integration of research knowledge and effective interventions into routine practice [17], holds promise for addressing health inequities and health care gaps [18–20]. The intention to advance health equity may be implied through the goals of optimizing interventions to their delivery context and improving the implementation and sustainment of effective interventions, yet the field is in the early stages of formulating and articulating a more explicit, widespread equity focus. Implementation science is rife with dozens of frameworks and models used to identify and characterize determinants that hinder or support implementation, guide processes for implementing and sustaining interventions, and evaluate outcomes of implementation efforts [21]. An initial synthesis of frameworks and models in implementation science suggests health equity has been relatively muted in the conceptual foundations of the field [21]. Despite recent advances in the development, refinement, and application of equity-focused implementation frameworks and models [22, 23], greater integration of health equity into implementation research is still needed [18–20, 24–26].

Given the growing attention to health equity in many areas of research and practice, there is a need for equity-focused conceptual guidance to inform study design, measurement, and evaluation [27]. However, there is no clear synthesis of which frameworks and models have guided equity-focused chronic disease research and intervention implementation, or the characteristics thereof. Furthermore, conceptual guidance is often not integrated consistently into empirical research and there is a lack of guidance around best practices for applying health equity frameworks and models to guide research [28]. As such, our review seeks to fill this knowledge gap by exploring the literature to identify health equity frameworks and models applied to the study of chronic disease. Specifically, our study has three primary aims and a fourth, exploratory aim:

- Aim 1: identify health equity frameworks and models applied in chronic disease prevention and control studies.
- Aim 2: characterize these health equity frameworks and models (e.g., construct definitions, relationships between constructs) using inductive and deductive approaches.
- Aim 3: describe how these health equity frameworks and models are used in chronic disease prevention and control studies.
- Aim 4: explore opportunities to integrate health equity frameworks and models in implementation science for chronic disease prevention and control.

## Methods

### Study design

Our review broadly explores health equity frameworks and models (referred to as FM from here on) applied in the study of chronic disease prevention and control. Although scoping reviews follow a process similar to that of systematic reviews, the latter is more appropriate for synthesizing and evaluating evidence and to produce guidance and recommendations [28]. Our review does not attempt to make definitive statements on an evidence base, rather seeks to clarify key concepts and definitions in the literature, examine how research is conducted on a particular topic, and to identify knowledge gaps and opportunities for learning across fields, thus a scoping review is the appropriate approach [29]. Our methods are guided by recommendations for conducting scoping reviews [30–32]. We will follow reporting guidelines outlined in the Preferred Reporting Items for Systematic Reviews and Meta-Analyses extension for scoping reviews (PRIMSA-ScR) [33], supplemented by the PRISMA extension for equity-focused reviews (PRISMA-E) [34]. The protocol is registered with the Open Science Framework (OSF; registration DOI:10.17605/OSF.IO/SFVE6). Any modifications to the protocol will be documented in OSF. Prior to protocol registration, one author searched the Cochrane Database of Systematic Reviews, PROSPERO, and OSF to determine if similar reviews were already underway; no similar reviews were found. This protocol follows the reporting recommendations from the PRISMA statement for protocols (PRISMA-P; Additional file 1). The following sections describe the steps for conducting a scoping review.

#### Identify research questions

Our team developed and refined our research questions through an iterative, collaborative process. In-depth discussions among our multidisciplinary team representing expertise in various content and methodological areas in public health science, health services research, implementation science, and health equity research, guided initial conceptualization. We then identified and reviewed articles on health equity theoretical framework and model development [2, 4–6, 27, 35–57] to inform our search strategy. Additionally, our team consulted experts in health equity research, anti-racism work, implementation science, and chronic disease control (see “Acknowledgements” section).

Given the pervasive burden of chronic diseases, and the need to establish a bounded scope as is recommended for scoping reviews [31, 32], we focused on this specific area within the study of health equity. We also selected chronic disease prevention and control for pragmatic reasons (i.e., alignment with the priorities of funders supporting our team members, and expertise of research centers with which our team members are affiliated). The focus on FM allows for delineation of the conceptual underpinnings of identified health equity-focused studies and aids the clear identification of relevant constructs that are mobilized to advance health equity. FM can enhance the effectiveness of interventions, improve the interpretability of study findings, and offer clear structure for designing and carrying out a study [21]. However, such conceptual guidance is often underutilized or applied inconsistently [28, 58]. Thus, we arrived at the aforementioned study aims to identify and characterize health equity FM that have been used in chronic disease research and their applications in empirical studies. We will also explore opportunities for integrating these FM into implementation research and practice. This project is part of an effort to expand health equity content within an existing dissemination and implementation science models webtool [59].

#### Identify relevant studies

Health equity is increasingly a matter of concern in scientific inquiry. Conceptualizations of health equity abound, often invoking related terms such as health disparities, health inequalities, and social determinants of health. While these terms are distinct from health equity, they are sometimes used interchangeably or inconsistently across the literature. Our team reviewed equity-related terms and definitions from various sources to frame our conceptualization of health equity and delineate nuances between related terms. These terms (see Additional file 2) guide our inclusion and exclusion criteria listed in Table 1.

**Table 1:** Inclusion/exclusion criteria

Criteria	Include	Exclude
**Y**ear published	2010 to 2021	Before 2010
**Language**	English	Non-English
**Study type**	Empirical studies: intervention studies (RCTs, quasi-experimental, etc.); observational studies (cohort, case–control, cross-sectional, case-crossover, ecologic, case series, case reports); qualitative studies; ^a^reviews (systematic, scoping), meta-analyses Studies must pertain to humans; may be conducted in any country	Non-empirical studies (e.g., commentary or debate papers, editorials, letter to the editor) Study of animals, cells, or other non-human subjects
**Chro**nic disease (per **CDC list of chronic diseases and associated risk/prevention factors **[60, 61])	Primary, secondary, or tertiary prevention, screening, maintenance, treatment, and/or survivorship in any of these chronic conditions: heart disease, cancer, chronic lung disease, stroke, Alzheimer’s, diabetes, chronic kidney condition, obesity Chronic disease risk/prevention topics: physical activity, diet/ nutrition, alcohol use, tobacco use; include even if not referenced in connection to a specific chronic disease listed above Include across lifespan from birth to end of life (e.g., breastfeeding in the context of chronic disease prevention) Include other conditions not listed above if studied in conjunction with eligible chronic disease or prevention topic (e.g., HIV/AIDS and heart disease; diet/nutrition, obesity, and depression)	Any non-health related topic (e.g., management practices in tech firms, environmental sustainability study that does not mention human health) Condition not on CDC chronic disease list and not paired with a condition on the list (e.g., standalone studies of HIV/AIDS, multiple sclerosis, osteoporosis, depression) Prevention topic other than the four listed (e.g., sun protection, safe needle exchange)
**Health equity**	Authors may communicate *intentionality* to study health equity in one or more ways: • Using key search terms in a context relevant to health (e.g., equity, justice, social determinants, racism) • Having equity-relevant aims/hypotheses/research questions (e.g., evaluating the impact of a nutrition policy to improve equitable access to healthy school meals) • Involving affected communities to redistribute power more fairly (e.g., advocacy groups, neighborhood residents involved in obesity prevention study design and execution) • Intervening to eliminate or overcome social or structural barriers to better health (e.g., multi-level intervention to improve accessibility of physical environment for people with mobility limitations) • Targeting an intervention towards a historically marginalized population and designing/adapting it to meet their needs/preferences (e.g., adapting a cancer screening program to improve access, linguistic and cultural concordance among rural migrant farmworkers) • Studying disparities affecting a historically marginalized population and a structural determinant of the disparity (e.g., education policy examined as a potential cause of asthma disparities in Black vs. White populations)	Relevant terms used in a different context (e.g., discrimination in measurement) Eligible chronic disease or prevention topic *without* a health equity focus Equity mentioned in secondary nature (e.g., health equity implications only in mentioned as future directions) Study within a historically marginalized population *without* consideration of health equity or related concepts (e.g., scale out of a cancer screening intervention tested in a high SES suburban population to a rural migrant farmworker population without adapting to better fit needs) Disparities studies that do not consider potential or known causes (e.g., type 2 diabetes prevalence by race/ethnicity stratified by SES, *without* assessing causes of disparities, such as racial residential segregation or discrimination) Disparities studies that examine individual-level manifestations of a structural cause of inequity (e.g., correlating individual educational attainment with asthma disparities in Black vs. white populations)
**Health equity framework or model (FM; added at full-text screening)**	Study describes or visually displays (via table, figure, or image) a FM that includes constructs conceptualized as being related to health equity AND there is clear application of the health equity FM (e.g., FM concepts appear in study aims, identification of intervention target, design of intervention components, target population and sampling, co-creation of interventions or research studies with impacted communities, selection of equity-relevant objectives, metrics, or outcomes; authors describe how FM was incorporated into study)	No FM referenced; FM not related to health equity; term used in a different context (e.g., statistical model) FM referenced, but not operationalized in the study (i.e., no clear application of the FM in aims, intervention components, sampling, measurement, or selected outcomes)

^a^review studies that meet all criteria at the title and abstract screening phase will be included for full-text review. Only review studies that apply a health equity FM to evaluate empirical studies included in the review sample will be eligible for inclusion for data extraction

##### Search strategy

Out team’s medical research librarian (BS) created search hedges containing terms related to ‘frameworks and models’, ‘chronic diseases and risk factors’ and ‘health equity or social determinants of health’. The chronic diseases and associated risk/prevention factors were those defined by the US Centers for Disease Control and Prevention (CDC) [60, 61]. The librarian conducted a systematic literature search in four bibliometric databases: PubMed (US National Library of Medicine), CINAHL + (Cumulative Index to Nursing and Allied Health Literature), APA PsycInfo (EBSCO), and Embase, with database-specific limiters applied (see Additional file 3 for search strategy).

##### Inclusion and exclusion criteria

Table 1 lists the inclusion and exclusion criteria, along with examples to illustrate how these are operationalized. We include peer-reviewed journal articles from any country, published in English between 2010 and 2021. This period reflects the substantial uptick in the appearance of health equity and related terms in publications starting in the 2010s [25]. Eligible study designs include intervention (e.g., randomized and quasi-experimental trials), observational (e.g., cohort, case–control, cross-sectional), or qualitative (e.g., ethnographic). We include review studies (e.g., scoping, systematic) and meta-analyses at title and abstract screening. Studies must pertain to one or more chronic conditions or prevention topics, as specified by the CDC (e.g., cancer, diabetes, cardiovascular disease), at any point along the prevention and control continuum [60, 61]. Studies must convey or describe *intentionality* around investigating health equity or related concepts (e.g., health justice); this may be reflected in equity-related study aims or in other ways described in Table 1. Studies of *health disparities* must either intervene on a causal factor beyond the individual level to reduce/eliminate the disparity, or examine structural determinants to identify an intervention target. For example, a study that observes disparities in type 2 diabetes prevalence between Black vs. white populations must intervene on an upstream determinant (e.g., improve the neighborhood food environment), or investigate a potential structural driver to explain the observed disparity or identify an upstream target for intervention (e.g., racial residential segregation and neighborhood food environment studied as potential causes that can be intervened upon to reduce disparities).

As many abstracts may lack information about FM, we apply the criterion related to health equity FM only during full-text screening. We define a health equity FM as a set of ideas, constructs, or variables arranged in a conceptual structure to guide or support the study of health equity. FM may be displayed visually (e.g., image, figure) or textually (e.g., table, constructs defined and described in text). Empirical articles must include a health equity FM and make explicit how the FM is integrated (e.g., FM informs the study aims, target outcomes, measurement). For review articles and meta-analyses, we will determine if the study applies a health equity FM to evaluate or characterize the original empirical articles in their sample; if a review does not apply a health equity FM in this way, we will exclude it. We will hand search references for original empirical studies reported in reviews for potentially relevant records to add to our review.

We exclude studies published prior to 2010, written in a language other than English, or that have irretrievable records. Non-empirical articles (e.g., editorials, conceptual or debate papers), conference abstracts, grey literature, or studies conducted among non-human subjects are ineligible. We exclude studies that do not examine a relevant chronic condition or prevention/risk factor. We exclude records not relevant to health equity, or that use equity-related terms in a different context. We exclude articles that do not convey intentionality around studying health equity or related concepts, or those that focus only on describing the presence of health disparities without assessing or intervening on the causes of disparities. For example, an observational study of asthma prevalence in minoritized racial groups compared to a reference group, controlling for socioeconomic status, that does not attribute observed disparities to structural causes (e.g., poor living conditions due to unjust housing policies) would be excluded.

#### Study selection

We will use EndNote reference management software to store records and conduct initial deduplication [62], followed by additional automated deduplication in the Covidence review management platform [63]. Given our initial bibliometric searches yielded over 65,000 records, our team will employ a single reviewer screening process at the title and abstract screening phase to enhance the feasibility of screening a large volume of records. An evaluation of single screening approaches found loss of sensitivity is minimized when such approaches are employed by experienced reviewers using clear screening guidance [64]. We will follow a robust, detailed screening protocol that incorporates several approaches supported by review methodology studies to enhance the rigor of our single screener methods. These steps include.

- Using a protocolized training led by authors with expertise in systematic and scoping review methods [64–66]
- Screening conducted by experienced reviewers who were involved in defining the review aims, scope, and screening criteria [64–66]
- Pilot screening to establish interrater reliability (IRR) prior to independent screening [65]
- Regular IRR checks throughout the title and abstract screening phase with stopping criteria if satisfactory IRR is not maintained [67]
- Use of Covidence’s machine learning capabilities to sort records by relevance and text mining to identify records with clear reasons for exclusion (e.g., animal studies, health topics not related to chronic disease), or that contain multiple relevant search terms [68–70]
- Team discussion of records for which a single screener cannot confidently come to a clear decision regarding eligibility [65]

During the pilot title and abstract screening, four reviewers will independently screen randomly selected sets of 20 records, meet to generate consensus, and refine the screening procedures. This process will repeat until a free-marginal multi-rater kappa of 0.8, indicating substantial agreement [71], is reached in two sequential screening rounds. The free-marginal multi-rater kappa is preferred to Fleiss’ kappa, as Fleiss’ kappa is intended for fixed rating rather than free rating (the latter is the approach for assigning eligibility in reviews), is prone to bias, and leads to the paradox of a low kappa value when absolute percent agreement among reviewers is high [71]. We will also calculate absolute percent agreement as an additional check for satisfactory IRR.

Following the pilot, reviewers will independently screen titles and abstracts in Covidence. If a reviewer is unable to decide, they will flag the record for discussion and the screening team will generate consensus. In cases of ambiguity or uncertainty, the team will include the record. The screening team will engage in biweekly IRR checks using the approach described in the pilot phase above. If the team does not maintain satisfactory IRR (kappa ≥ 0.8), we will pause independent screening, repeat the pilot procedures, and resume independent screening only when satisfactory IRR is achieved. We anticipate these procedures will result in approximately 1000 records being reviewed by the full screening team. We will document consensus decisions from the pilots, IRR checks, and group discussions so screeners can refer to these to inform independent screening decisions.

Upon completing title and abstract screening, we will pilot the full-text screening procedures with a randomly selected set of 20 articles and generate consensus. The screening team will refine procedures and conduct additional pilots as needed. After the pilot, reviewer pairs will conduct blinded dual independent screening, such that all full-text records will be screened by two reviewers. Reviewers will apply a hierarchical exclusion coding process in which they will code the highest-order applicable exclusion reason (e.g., reviewers will code ineligible study design before no health equity FM if both reasons apply). We will not code multiple reasons for exclusion. Reviewer pairs will meet regularly to discuss disagreements and generate consensus. If the pair cannot achieve consensus, the screening team will discuss and make a final decision. For records included at the full-text phase, we will check citations for the relevant health equity FM and hand search FM development articles, as applicable, to supplement the empirical articles located through our search. For review studies and meta-analyses, we will hand search the references for original empirical studies not located in our bibliometric searches to screen for inclusion in our review.

#### Data extraction

The team will develop a data extraction codebook and Covidence extraction database. The codebook will list the data elements to be extracted from articles, provide definitions for each data element, elucidate coding rules, and provide examples. The extraction database will contain free-text cells to enter verbatim text from articles to be coded inductively, and fixed response codes to deductively categorize article data, as applicable. The database will also include a notes field to record other potentially relevant information or reflections that may inform development of inductive codes, interpretation of the results, or notable discussion points (e.g., potential relevance to implementation science). We will pilot the extraction and quality assessment procedures with a subset of 5 randomly selected articles, refine the procedures and database, and conduct additional piloting as needed.

We will use a dual non-independent consensus coding approach successfully applied in other reviews conducted by our team members [72–76]. A primary reviewer will extract relevant information and enter it into the database. A secondary coder will review the data extraction for accuracy and completeness, flag any discrepancies, and note recommended changes. We will apply the Mixed Methods Appraisal Tool (MMAT) to evaluate study quality during extraction [77]. The MMAT contains objective methodology-specific rating criteria and provides guidance on qualitative assessments of study strengths and limitations, serving as a valuable tool for drawing comparisons across studies and identifying trends. Coders will meet to discuss disagreements, generate consensus, and record final decisions in the extraction database. Coders will consult the screening team for a final decision if the pair cannot reach consensus. Data extraction will include, but will not be limited to:

- Article bibliometric information: author, publication year, journal name
- Study context: country, setting type (e.g., clinical, community), target population characteristics, chronic disease topic, intervention name and description (if applicable)
- Study design: methodology, data collection, health-equity related variables, focus of primary outcomes (e.g., individual health, population health, contextual assessment, formative evaluation, implementation)
- FM characteristics: FM name, original citation (if development is described in another article), socioecological level(s) conceptualized in the FM (e.g., intrapersonal, interpersonal, organization, community, policy), equity-relevant constructs, definition of health equity and related terms, and relationships between constructs (e.g., linear, nested, cyclical, feedback loops)
- FM uses: study components to which FM are applied (e.g., inform study aims/research questions, intervention design, identification of target population, sampling and recruitment approach, selection of outcomes, measurement), degree of FM integration into the study (e.g., low, moderate, high)

#### Collating, summarizing, and reporting the results

We will report the search yields and number of records included at each phase per the PRISMA-ScR [33]. We will quantitatively summarize characteristics of our sample (e.g., frequencies and proportions of types of study designs, countries and settings in which studies were conducted, target population characteristics, health topics). We will report these data in tables and summarize narratively in text. For FM characteristics, we will quantitively summarize the total number of unique FM in our sample, total number of empirical uses of each FM in the sample, and calculate frequencies and proportions for the FM characteristics (e.g., FM types, nature of relationships between constructs). We will narratively describe health equity conceptualizations and summarize themes across studies. We will summarize qualitative themes related to potential applications of health equity FM to implementation science (e.g., use of FM to characterize contextual determinants, selecting equity-related implementation outcomes). We will also compile case examples of FM utilization, including descriptions of how health equity FM are applied within implementation research.

#### Consultation

Each stage of our review involves consultation with an array of collaborators. The team consulted with external experts to inform the conceptualization of our study and creation of the search strategy. Coders will meet with the broader study team to provide updates, share findings, and gather feedback. We will provide works in progress updates to scientific experts to gather feedback on the review process and interpretation of the results. We will share preliminary findings via institutional, regional, national, and international research interest group meetings, conferences, and other interactive dissemination outlets [78]. These inputs will help ensure findings are understandable and relevant to our target audience and will generate insights for interpreting and contextualizing our results.

### Study team positionality

Our team is comprised of members from numerous health-related disciplines, spanning several professional roles and stages of career. We hold multiple intersecting identities and roles in academic research and social justice commitments, spanning myriad racial identities, ethnic, cultural, social, socioeconomic and family backgrounds, gender identities, sexual orientations, physical and mental abilities, religious affiliations, and countries of origin. Collectively, we bring a wealth of personal and professional experiences to the study of health equity, and draw upon these experiences to strengthen the work of this team.

Our team members, though currently all located in the USA, represent nationalities spanning four continents (Africa, Europe, North America, and South America), with diverse engagements with global health perspectives. We recognize the limits of our perspectives as scholars at well-resourced US-based academic institutions and attempt to direct the power and advantage afforded by our educational and professional opportunities toward advancing health equity. While we hold power, our team members are predominantly trainees (graduate students, postdoctoral fellows) and early and mid-career non-tenured faculty, thus hold relatively vulnerable status compared to senior scholars. We actively seek out different perspectives to challenge, balance, and learn from the assumptions each of us bring to the study of health equity. We also recognize the nature of this study is biased towards the inclusion of literature written in English published in peer-reviewed journals indexed in scientific databases, which tend to skew towards high-income countries, and often do not offer a representative account of the perspectives of directly affected communities. Acknowledging these limitations affirms the need for efforts toward making scientific equity more explicit and accessible for peer-review and shaping critical knowledge structures to achieve health equity [12].

## Discussion

This scoping review seeks to map rapidly growing efforts to incorporate an explicit health equity focus into public health, health services, and implementation research, specifically along the chronic disease prevention and control continuum. The primary goal of this review is to identify and characterize health equity FM that have been utilized in chronic disease research since 2010. Additionally, we explore implications for health equity FM utilization within the field of implementation science, and seek to identify gaps and elucidate future opportunities for equity-focused research. The findings from this review will offer researchers and practitioners interested in incorporating health equity FM into their work a collated list of FM, descriptions of FM characteristics, current applications in empirical studies, and suggestions for future uses. We plan to disseminate health equity FM with potential relevance to implementation science and accompanying application case studies through an existing open-access resource, the Dissemination and Implementation Models in Health Research and Practice webtool [59], which aids implementation researchers and practitioners in identifying and incorporating FM into research studies or implementation projects.

The methods employed in this review seek to balance rigor and efficiency to accommodate a high record yield. We follow established guidelines for conducting scoping reviews [30–32]; any deviations will be carefully planned, monitored, and reported in our OSF registration and publications disseminating our findings. We plan to use a single-reviewer approach at the title and abstract screening phase, which has potential limitations in terms of reviewer sensitivity (i.e., excluding records that should be included) and selection bias. We incorporate several practices, including use of detailed protocols, reviewer training, regular consensus discussions and IRR checks, and use of Covidence’s machine learning and text mining capabilities, to improve the rigor of this approach [63–69]. Findings from the review methodology literature suggest combining multiple screening support methods such as the ones employed in this review can minimize bias and loss of sensitivity [64, 70] and have been successfully utilized in other reviews conducted by the authors [76, 79]. Our study could offer a useful example to other researchers seeking to enhance the rigor of a single screener approach in rapid evidence syntheses or to feasibly screen a high volume of records.

Other limitations of this review are the exclusion of articles published prior to 2010 in languages other than English. This biases the sample towards studies conducted in English speaking countries and could exclude relevant FM and insights that have not yet been realized in more recent studies. Further, the exclusion of non-empirical articles and non-peer reviewed sources may omit FM described in conceptual articles, books, and other sources. We plan to conduct hand searches of the FM citations from empirical studies included in our review to locate the original development articles first describing these FM. We will add these development articles to extraction, even if the development article is published before 2010 or is non-empirical. It is the intent of this review to identify health equity FM used in empirical studies of chronic disease prevention and control. Thus, FM only described conceptually or in other areas of health (e.g., infectious disease, mental health) are beyond the bounds of this scoping review and could be explored and compared in future work.

Our team incorporated a collaborative approach in the conceptualization of this review and will continue to engage other relevant groups throughout the study stages. We draw upon input from a wide network of researchers representing an array of expertise in implementation science, health equity and chronic disease research. While this is a considerable strength of our study, currently this is limited to mostly US-based academic collaborators. We endeavor to gather input from and share findings with colleagues outside the USA and non-academic audiences as the project continues. We plan to disseminate findings through multiple channels, including peer-reviewed journals, conference presentations, webinars, and open-access websites. The detailed documentation of our methods and transparency in reporting will provide opportunities for other researchers to replicate, innovate, and expand upon this work in additional health content areas. Findings from this review are expected to inform knowledge production that improves the health equity impact of chronic disease prevention and control research in public health and implementation science.

## Supplementary Information

**Additional file 1.** PRISMA-P (Preferred Reporting Items for Systematic review and Meta-Analysis Protocols) 2015 checklist: recommended items to address in a systematic review protocol*

**Additional file 2.** Health equity and related terms and definitions [80–89].

**Additional file 3: Appendix 2.** Search Strategy

## Data Availability

Data sharing is not applicable to this article as no datasets were generated or analyzed for the purposes of this publication.

## Abbreviations

CDC: US Centers for Disease Control and Prevention
FM: Framework(s) and model(s)
IRR: Interrater reliability
OSF: Open Science Framework
PRISMA: Preferred Reporting Items for Systematic Reviews and Meta-Analyses
PRISMA-E: PRISMA Equity Extension
PRISMA-P: PRISMA Extension for Review Protocols
PRISMA-ScR: PRISMA Extension for Scoping Reviews
RCT: Randomized control trial
WHO: World Health Organization
