# Supplementary material for: Protocol for a scoping review of health equity frameworks and models applied in empirical studies of chronic disease prevention and control
Source: Syst Rev. 2023 May 11;12:83. doi: 10.1186/s13643-023-02240-2 (PMC10176929; doi:10.1186/s13643-023-02240-2)
Supplement: Supplementary file 2 — Additional file 2. Health equity and related terms and definitions [80–89]. [file 13643_2023_2240_MOESM2_ESM.docx]

**Additional File 2: Health equity and related terms and definitions**

| **Term** | **Definition** |
| --- | --- |
| Health Equity | Jones, 2014: “…assurance of the conditions for optimal health for all people. Achieving health equity requires valuing all individuals and populations equally, recognizing and rectifying historical injustices, and providing resources  according to need. Health disparities will be eliminated when health equity is achieved.” Health equity is conceived as a process that involves “active inputs, constant vigilance, and continuous correction.” [5]  Braveman, 2017: “everyone has a fair and just opportunity to be as healthy as possible. This requires removing obstacles to health such as poverty, discrimination, and their consequences, including [disenfranchisement] and lack of access to good jobs with fair pay, quality education and housing, safe environments, and health care.” For the purposes of measurement, health equity means reducing and ultimately eliminating disparities in health and its determinants that adversely affect excluded or marginalized groups [6] |
| Health Disparities/ Inequalities | Braveman, 2006, 2014: used interchangeably, the terms refer to potentially avoidable health differences closely linked with economic, social, or environmental disadvantage; may be used as a metric for measuring progress towards achieving health equity, but are not synonymous with health equity [34, 42] |
| Social Determinants of Health | Alderwick & Gottlieb, 2019, WHO, 2008: “the conditions in which people are born, grow, live, work and age, which are shaped by the distribution of money, power and resources” [80, 81]  Jones, 2009: “the contexts of our lives; determinants of health outside of the individual, beyond individual behaviors and individual genetic endowment. These contexts are not randomly distributed, they are shaped by historical injustices and contemporary structural factors that perpetuate the historical injustices” [2] |
| Structural Determinants of Health (inequities) | Solar & Irwin, 2010: “social processes underlying the unequal distribution of factors [that promote or undermine health] between groups occupying unequal positions in society…” and refers to the “interplay between the socioeconomic-political context, structural mechanisms generating social stratification and the resulting socioeconomic position of individuals”; also referred to as “root causes of inequities.” [82] |
| Historically Marginalized Population | Hall et al., 1994: Marginalization occurs when “persons are peripheralized based on their identities, associations, experiences, and environment.” [83]  Marginalization is context-dependent, may shift over time or by place, and may be experienced in different ways by varying intersecting identities. The following list is not exhaustive, but offers a foundation for considering characteristics that may be subject to marginalization: race, ethnicity, gender identity, sexual orientation, socioeconomic status (SES), housing status, geographic region, physical or mental ability, nationality, immigration status, language, literacy, religion, history of abuse, currently or formerly incarcerated people and their families [5, 83-89] |
