# Supplementary material for: Protocol for a scoping review of health equity frameworks and models applied in empirical studies of chronic disease prevention and control
Source: Syst Rev. 2023 May 11;12:83. doi: 10.1186/s13643-023-02240-2 (PMC10176929; doi:10.1186/s13643-023-02240-2)
Supplement: Supplementary file 3 — Additional file 3: Appendix 2. Search Strategy [file 13643_2023_2240_MOESM3_ESM.docx]

**Appendix 2: Search Strategy**

Limiters: PY = 2010-current; human studies only/no animal; English language; abstract available; journal article only

Databases searched: PubMed, Embase, CINAHL+, APA PsycInfo

TOTAL RECORDS FOR ALL DATABASES: 90,975. After deduplication in Endnote = 65,547

| **Action** | **Term** | **Records Returned** |
| --- | --- | --- |
| 1 | ("Health Policy"[MeSH Major Topic] OR "Concept Formation"[MeSH Terms] or "models, theoretical"[MeSH Terms]) with limiters | 69,516 |
| 2 | (framework*[Title/Abstract] OR concept*[Title/Abstract] OR pathway[Title/Abstract] OR approach*[Title/Abstract] OR constructs[Title/Abstract] OR blueprint[Title/Abstract] OR model*[Title/Abstract]) OR (framework*[Text Word] OR concept*[Text Word] OR pathway[Text Word] OR approach*[Text Word] OR constructs[Text Word] OR blueprint[Text Word] OR model*[Text Word]) with limiters | 397,595 |
| 3 | 1 or 2 | 399,728 |
| 4 | ("Social Determinants of Health"[MeSH Terms] OR "Health Status Disparities"[MeSH Terms] OR "Health Status"[MeSH Terms]) with limiters | 49,216 |
| 5 | ("health equit*"[Title/Abstract] OR "health equalit*"[Title/Abstract] OR "health inequit*"[Title/Abstract] OR "health inequalit*"[Title/Abstract] OR "health disparit*"[Title/Abstract] OR "justice"[Title/Abstract]) OR ("health equit*"[Text Word] OR "health equalit*"[Text Word] OR "health inequit*"[Text Word] OR "health inequalit*"[Text Word] OR "health disparit*"[Text Word] OR "justice"[Text Word]) with limiters | 4,701 |
| 6 | (racism[Title/Abstract] OR discrimination[Title/Abstract] OR "sdoh"[Title/Abstract] OR segregation[Title/Abstract] OR colonialization[Title/Abstract] OR "critical race"[Title/Abstract]) OR (racism[Text Word] OR discrimination[Text Word] OR "sdoh"[Text Word] OR segregation[Text Word] OR colonialization[Text Word] OR "critical race"[Text Word]) with limiters | 5,821 |
| 7 | Or/4-6 | 57,709 |
| 8 | 3 AND 7 | 86,281 |
| 9 | ("Chronic Disease"[Mesh] OR "Chronic Disease Indicators"[Mesh] OR "Noncommunicable Diseases"[Mesh]) with limiters | 15,024 |
| 10 | ("chronic disease*"[Title/Abstract] OR "chronic illness"[Title/Abstract] OR "chronically ill"[Title/Abstract] OR "chronic disease indicator*"[Title/Abstract] OR "chronic disease surveillance"[Title/Abstract] OR "noncommunicable disease*"[Title/Abstract] OR "non-infectious disease*"[Title/Abstract]) OR ("chronic disease*"[Text Word] OR "chronic illness"[Text Word] OR "chronically ill"[Text Word] OR "chronic disease indicator*"[Text Word] OR "chronic disease surveillance"[Text Word] OR "noncommunicable disease*"[Text Word] OR "non-infectious disease*"[Text Word]) with limiters | 24,382 |
| 11 | ("Coronary Disease"[Mesh] OR "Heart Disease Risk Factors"[Mesh] OR "Heart Diseases"[Mesh] OR "Heart Diseases"[Majr] OR "Stroke"[Majr] OR "Cardiovascular Diseases"[Mesh] OR “aneurysm”[Mesh]) with limiters | 151,306 |
| 12 | ("heart disease*"[Title/Abstract] OR "vascular disease*"[Title/Abstract] OR "high blood pressure"[Title/Abstract] OR "hypertension"[Title/Abstract] OR "aneurysm"[Title/Abstract] OR "stroke"[Title/Abstract]) OR ("heart disease*"[Text Word] OR "vascular disease*"[Text Word] OR "high blood pressure"[Text Word] OR "hypertension"[Text Word] OR "aneurysm"[Text Word] OR "stroke"[Text Word]) with limiters | 84,293 |
| 13 | ("Neoplasms"[Mesh] OR "Early Detection of Cancer"[Mesh]) with limiters | 208,319 |
| 14 | ("cancer diagnosis"[Title/Abstract] OR "cancer screen*"[Title/Abstract] OR "cancer test*"[Title/Abstract] OR "cancer*"[Title/Abstract] OR "neoplasm*"[Title/Abstract]) OR ("cancer diagnosis"[Text Word] OR "cancer screen*"[Text Word] OR "cancer test*"[Text Word] OR "cancer*"[Text Word] OR "neoplasm*"[Text Word]) with limiters | 221,225 |
| 15 | ("Pulmonary Disease, Chronic Obstructive"[Mesh] OR "Lung Diseases, Obstructive"[Mesh]) with limiters | 14,850 |
| 16 | ("asthma"[Title/Abstract] OR "COPD"[Title/Abstract] OR "chronic obstructive pulmonary disease"[Title/Abstract]) OR ("asthma"[Text Word] OR "COPD"[Text Word] OR "chronic obstructive pulmonary disease"[Text Word]) with limiters | 19,227 |
| 17 | "Alzheimer Disease"[Mesh] OR "Dementia"[Mesh] with limiters | 17,300 |
| 18 | ("dementia"[Title/Abstract] OR "senile dementia"[Title/Abstract] OR "alzheimer*"[Title/Abstract]) OR ("dementia"[Text Word] OR "senile dementia"[Text Word] OR "alzheimer*"[Text Word]) with limiters | 25,930 |
| 19 | ("Diabetes Mellitus"[Mesh] OR "Diabetes Mellitus, Type 1"[Mesh] OR "Diabetes Mellitus, Type 2"[Mesh]) with limiters | 41,405 |
| 20 | ("diabetes"[Title/Abstract] OR "juvenile diabetes"[Title/Abstract] OR "type 1 diabetes"[Title/Abstract] OR "type 2 diabetes"[Title/Abstract]) OR ("diabetes"[Text Word] OR "juvenile diabetes"[Text Word] OR "type 1 diabetes"[Text Word] OR "type 2 diabetes"[Text Word]) with limiters | 60,618 |
| 21 | ("Chronic Kidney Disease-Mineral and Bone Disorder"[Mesh] OR "Renal Insufficiency, Chronic"[Mesh]) with limiters | 10,886 |
| 22 | ("chronic kidney disease*"[Title/Abstract] OR "chronic renal disease*"[Title/Abstract] OR "chronic renal insufficiency"[Title/Abstract] OR "kidney disease*"[Title/Abstract]) OR ("chronic kidney disease*"[Text Word] OR "chronic renal disease*"[Text Word] OR "chronic renal insufficiency"[Text Word] OR "kidney disease*"[Text Word]) with limiters | 16,588 |
| 23 | ((obesity[Text Word] OR obese[Text Word] OR overweight[Text Word] OR weight[Text Word] OR BMI[Text Word] OR "body mass index"[Text Word] OR exercise[Text Word] OR "physical activity"[Text Word] OR "physical inactivity"[Text Word] OR "physical fitness"[Text Word] OR sedentary[Text Word] OR lifestyle[Text Word] OR diet*[Text Word] OR "dietary intake"[Text Word] OR "food intake"[Text Word] OR "healthy eating"[Text Word] OR nutrition*[Text Word] OR fruit*[Text Word] OR vegetable*[Text Word] OR food*[Text Word] OR "soft drink*"[Text Word] OR soda[Text Word] OR sweetened[Text Word] OR sugar*[Text Word] OR smok*[Text Word] OR "smoking cessation*"[Text Word] OR tobacco[Text Word] OR cigarette[Text Word] OR nicotine[Text Word] OR vape*[Text Word] OR "vaping"[Text Word] OR "e-cig*"[Text Word] OR "electronic cigarette"[Text Word] OR pipe[Text Word] OR cigar[Text Word] OR hookah[Text Word] OR alcohol*[Text Word] OR drink*[Text Word] OR drunk*[Text Word] OR liquor*[Text Word] OR intoxicat*[Text Word])) OR (obesity[Title/Abstract] OR obese[Title/Abstract] OR overweight[Title/Abstract] OR weight[Title/Abstract] OR BMI[Title/Abstract] OR "body mass index"[Title/Abstract] OR exercise[Title/Abstract] OR "physical activity"[Title/Abstract] OR "physical inactivity"[Title/Abstract] OR "physical fitness"[Title/Abstract] OR sedentary[Title/Abstract] OR lifestyle[Title/Abstract] OR diet*[Title/Abstract] OR "dietary intake"[Title/Abstract] OR "food intake"[Title/Abstract] OR "healthy eating"[Title/Abstract] OR nutrition*[Title/Abstract] OR fruit*[Title/Abstract] OR vegetable*[Title/Abstract] OR food*[Title/Abstract] OR "soft drink*"[Title/Abstract] OR soda[Title/Abstract] OR sweetened[Title/Abstract] OR sugar*[Title/Abstract] OR smok*[Title/Abstract] OR "smoking cessation*"[Title/Abstract] OR tobacco[Title/Abstract] OR cigarette[Title/Abstract] OR nicotine[Title/Abstract] OR vape*[Title/Abstract] OR "vaping"[Title/Abstract] OR "e-cig*"[Title/Abstract] OR "electronic cigarette"[Title/Abstract] OR pipe[Title/Abstract] OR cigar[Title/Abstract] OR hookah[Title/Abstract] OR alcohol*[Title/Abstract] OR drink*[Title/Abstract] OR drunk*[Title/Abstract] OR liquor*[Title/Abstract] OR intoxicat*[Title/Abstract]) | 224,180 |
| 24 | Or/9-23 | 617,208 |
| 25 | 8 AND 24 | 11,507 |

Note: Search conducted November 4, 2021
